# Seasonal Dynamics of Red Imported Fire Ant (*Solenopsis invicta*) Colony Structures Across *Camellia oleifera* Plantations and Fishponds in South China

**DOI:** 10.3390/ani15101483

**Published:** 2025-05-20

**Authors:** Yuling Liang, Jingxin Hong, Yunbo Song, Kuo Yue, Meng Chen, Jiarui Wu, Yangting Ou, Mingrong Liang, Yongyue Lu

**Affiliations:** 1 Red Imported Fire Ant Research Center, South China Agricultural University, Guangzhou 510642, China; liangyuling@stu.scau.edu.cn (Y.L.); hongjingxin@stu.scau.edu.cn (J.H.); songyunbo-scau@stu.scau.edu.cn (Y.S.); fnyuekuo@163.com (K.Y.); 19877989670@163.com (M.C.); wujiarui@stu.scau.edu.cn (J.W.); oyting@stu.scau.edu.cn (Y.O.); 2 Insect Biodiversity and Biogeography Laboratory, School of Biological Sciences, The University of Hong Kong, Pok Fu Lam Road, Hong Kong SAR, China

**Keywords:** *Solenopsis invicta*, agro-ecosystems, population dynamics, colony structure, caste proportion, environmental factors, correlation

## Abstract

The red imported fire ant (*Solenopsis invicta*, RIFA) is a globally invasive species with strong sensitivity to environmental conditions. This study investigated the seasonal dynamics and colony structure of RIFA over the course of one year across two typical habitats in South China: *Camellia oleifera* plantations and fishponds. The results revealed clear seasonal patterns in caste composition. Worker abundance peaked during winter (December–January), while reproductive individuals (queens, males, and alates) emerged primarily in spring and early summer (March–May). Colony biomass, worker number, and individual dry weight were significantly higher in *C. oleifera* plantations, whereas fishpond habitats exhibited greater numbers of larvae and male alates, suggesting different reproductive allocation strategies across habitats. An analysis of caste composition indicated that adult workers were dominant in both habitats, but the proportion of pupae was notably higher in fishpond colonies, especially in spring. Significant correlations were found between colony metrics and nest characteristics, including a negative relationship between worker body length and colony biomass. Environmental factor analysis showed that air pressure positively influenced worker numbers, while temperature was negatively associated with them. Precipitation and humidity played key roles in regulating larval and pupal populations. Overall, *RIFA* exhibited strong seasonal patterns and ecological plasticity in response to habitat differences and environmental variables. These findings provide insights into the species’ invasion biology and inform habitat-specific monitoring and management strategies.

## Simple Summary

The red imported fire ant (*Solenopsis invicta*, RIFA) is an aggressive and invasive insect that poses ecological and economic threats in many parts of the world. Understanding how these ants behave in different environments and seasons is important for controlling their spread. In this study, we examined the colony structure of RIFA in two common habitats in South China—*Camellia oleifera* plantations and fishponds—over the course of one year. We found that the number of worker ants peaked in winter, while reproductive ants (such as queens and winged males and females) appeared mostly in spring and early summer. Colonies in *C. oleifera* plantations were larger and had heavier workers, while fishpond colonies had more larvae and male ants. Environmental factors like air pressure, temperature, and rainfall influenced ant development in different ways depending on the habitat. These findings help explain how RIFA adapts to its environment and may assist in developing better ways to monitor and manage this invasive species.

## 1. Introduction

The red imported fire ant (*Solenopsis invicta*, RIFA) is a globally invasive species that is notable for its complex colony structure, biological traits, and behavioral characteristics [1,2,3,4]. Its life cycle comprises four stages—egg, larva, pupa, and adult—with developmental time and lifespan closely linked to body size [5]. Worker ants exhibit size-dependent lifespans, while the reproductive capacity of the queen directly influences colony growth and expansion [6]. The colony structure of RIFA is highly organized, with distinct social roles among workers and reproductive ants [5]. This division of labor extends to their behavioral patterns and environmental adaptability. Colonies consist of queens, reproductive males, reproductive females, and workers, with workers accounting for over 95% of the population [7,8]. These workers are responsible for foraging, brood care, nest construction, and defense [4,7].

The colony structure and differentiation of RIFA play a crucial role in its development and local expansion. Previous studies have provided comprehensive insights into colony size, caste composition, and population regulation mechanisms, highlighting their significance in shaping RIFA’s ecological success and invasion dynamics [9,10]. Colony size peaks during the warmest seasons, ranging from 57,000 to 187,000 individuals, while moderate temperatures result in smaller colonies of 14,000 to 103,000 individuals. These findings underscore the strong correlation between environmental temperature and colony dynamics. In addition to thermal influences, the invasion success of RIFA has been linked to biological traits such as polygyny, colony budding, aggressive foraging, and high reproductive capacity, which together enhance its competitiveness and adaptability in novel environments [11,12,13].

In southern China, RIFA exhibits distinct seasonal activity patterns [4,14]. Activity peaks typically occur from April to June, with a secondary peak from September to November [15,16]. Seasonal activity is strongly influenced by temperature and humidity, with midday activity in winter and spring, and morning or evening activity in summer. Monthly average temperature, precipitation, and minimum temperature are key factors affecting population fluctuations, while precipitation and soil moisture significantly influence their biological traits, behavior, and nest-building activities [17,18]. Additionally, RIFA demonstrates remarkable drought tolerance, with its eco-physiological mechanisms playing a critical role in adapting to environmental changes [19].

Despite these insights, significant gaps remain in understanding the colony dynamics of RIFA. Most previous studies have relied on short-term observations, often lacking long-term monitoring needed to capture seasonal variation and adaptive responses. Although RIFA has been studied in habitats such as litchi orchards [20], banana plantations [21], urban greenbelts [16], and wastelands [22], its population dynamics in *C. oleifera* plantations and fishpond ecosystems remain largely unexplored. In particular, the interaction between colony structure and environmental drivers in these habitats is poorly understood, limiting our ability to predict invasion success and develop targeted control strategies. To address this knowledge gap, the present study focuses on *C. oleifera* plantations and fishpond habitats. We conducted systematic monthly surveys over a 13-month period to characterize the seasonal dynamics of RIFA colonies in these environments. Specifically, we quantified the seasonal composition of colony members across developmental stages—including workers, worker larvae, worker pupae, queens, alate females, alate males, alate larvae, and alate pupae—and examined variations in nest size. Environmental variables were integrated to explore the species’ adaptive mechanisms and patterns of colony regulation.

Understanding the dynamics of RIFA in *C. oleifera* plantations and fishpond ecosystems fills a critical knowledge gap in invasion ecology. Given the economic importance of both agricultural and aquacultural systems, this study provides timely insights into how RIFA colonies behave under distinct habitat conditions, contributing to efforts to reduce invasive impacts and protect sensitive production systems. Furthermore, characterizing seasonal patterns in ecologically sensitive fishponds offers a foundation for developing targeted, habitat-specific control strategies. These findings support a bottom-up management framework that empowers farmers and stakeholders to monitor and respond to infestation risks more effectively.

Therefore, the objective of this study is to characterize the seasonal dynamics of RIFA colony structure across two contrasting agro-ecosystems in South China and to evaluate how reproductive patterns and environmental factors shape colony development in each habitat.

## 2. Materials and Methods

### 2.1. Study Area and Habitats

**Figure 1:**
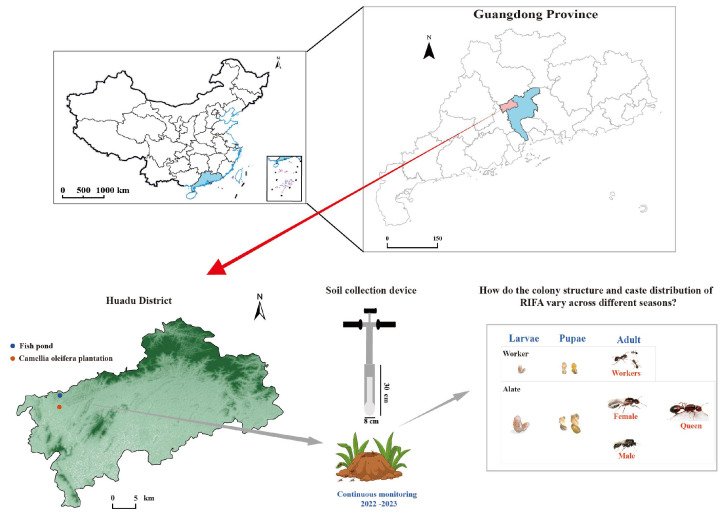
Schematic of sampling locations and sample collection to monitor the seasonal dynamics of the RIFA colony structure. Map of Huadu District, Guangdong province, China, showing the locations of the fishpond (blue solid circle) and *C. oleifera* plantation (red solid circle), where soil samples were collected for continuous monitoring of RIFA colonies from August 2022 to August 2023. A soil collection device (shown in the middle of Figure 1) was used to extract samples at a width of 8 cm and a depth of 30 cm (volume of approximately 1507 cm^3^) for studying RIFA colony structure and caste distribution. The map of China was obtained from the Standard Map Service website of the National Administration of Surveying, Mapping, and Geoinformation of China (http://bzdt.ch.mnr.gov.cn/index.html) (accessed on 1 April 2025), designated by review number GS (2023) 2767. It was converted into a Shapefile (SHP) format for further analysis and cartographic purposes.

The study was conducted in two habitats located near Meilin Lake in Huadu District, Guangzhou, China: a *C. oleifera* plantation (113.0382979° E, 23.46590357° N) and a fishpond system (113.0305088° E, 23.46419113° N) (Figure 1).

Three fixed plots, each covering over 2000 m^2^, were established in both the *C. oleifera* plantation and fishpond habitats. The 13-month survey, conducted from August 2022 to August 2023, recorded an average temperature of 23.55 °C, with the lowest temperatures occurring in December and the highest from June to September. Before the full-year monitoring began, a preliminary survey was conducted to assess baseline RIFA density in each habitat. Nest counts and infestation levels were determined based on the RIFA Monitoring Protocol (GB/T 23626-2009) [23,24].

Classification of Live Ant Nest Density per Unit Area:

To quantify live ant nest density, at least three random 667 m^2^ plots were selected in each habitat. The density of live nests per 100 m^2^ was classified into five levels based on the following criteria:

Level I (Mild): 0 to 0.1 live nests per 100 m^2^.

Level II (Moderate): 0.11 to 0.5 live nests per 100 m^2^.

Level III (Moderately Heavy): 0.51 to 1.0 live ant nests per 100 m^2^.

Level IV (Heavy): 1.1 to 10 live ant nests per 100 m^2^.

Level V (Severe): more than 10 live ant nests per 100 m^2^.

To track population dynamics and colony expansion, the number of RIFA nests and worker ant densities were recorded monthly throughout the study period.

### 2.2. Sampling and Measurement

Monthly surveys were conducted in the fishpond habitat and the *C. oleifera* habitat in Huadu District, Guangzhou, China. A standardized soil collection device was used to investigate the structure of RIFA colonies (Figure 1). Each month, 10 ant nests with a typical elliptical mound shape were randomly selected in each habitat for sampling. Before sampling, the external dimensions of each mound—length (*a*), width (*b*), and above-ground height (*ht*)—were measured. Prior to collecting soil samples, the inner edges of plastic boxes were coated with talc powder [25,26] to prevent the escape of RIFA. Soil samples were then extracted from the center of each mound using the same standardized soil collection device (Shaoxing Shangyu Yinghao Hardware Instruments Co., Ltd., Shaoxing, Zhejiang, China).

Only well-developed nests—defined as those with a clearly visible above-ground mound, active worker traffic, and the presence of brood (eggs, larvae, or pupae)—were selected for sampling. To ensure data consistency, only undamaged nests were sampled on sunny days. All samples were promptly collected and stored in plastic containers (20 cm × 30 cm × 10 cm) for subsequent laboratory analysis.

The size of each anthill was assessed by calculating its basal area (*S*) and volume (*V*) using the following formulas [22,25]:

$$S=\pi \times \frac{a}{2}\times \frac{b}{2}$$

$$V=\frac{4}{3\pi }\times \frac{a}{2}\times \frac{b}{2}\times ht$$

where *a* is the length, *b* is the width, and *ht* is the height of the anthill above the ground.

### 2.3. Colony Separation, Caste Identification, and Biomass Measurement

Utilizing the characteristic raft-forming behavior of RIFA colonies when exposed to water [27], we employed a water-dripping method to gradually saturate the soil and separate ants from the soil matrix [28]. This method takes advantage of the hydrophobic properties of the ants’ cuticles, which allow individuals to cluster into buoyant aggregates during flooding events [27]. The extracted colonies were preserved in anhydrous ethanol for subsequent analysis. After separation and drying, individual castes—including queens, reproductive females, males, larvae, and pupae—were identified and counted based on morphological characteristics under visual inspection. Eggs were excluded due to their small size and negligible contribution to overall colony metrics. The total number of worker ants was also estimated visually, and their collective dry biomass was measured using an electronic balance.

### 2.4. Source of Meteorological Data

Data were obtained from the National Weather Science Data Center (http://www.ruifengbio.com/products/zhihuinongye/1258.html) (accessed on 17 February 2024). Data are daily meteorological data from August 2022 to August 2023, including monthly humidity, precipitation, barometric pressure, and air temperature.

### 2.5. Statistical Analysis

Data were initially organized and processed using Microsoft Excel 2013. To visualize seasonal variation patterns, time series plots were generated in Origin 2021.

For more detailed analysis, colony structure, mound characteristics, and worker traits across different habitats were analyzed in R 4.1.2 using the ggplot2 package [29]. This included boxplots, circular percentage charts, scatterplots, and regression analyses to illustrate differences in colony composition, caste ratios, and the relationships between colony abundance and mound size.

Statistical analyses were conducted as follows:

Independent t-tests were conducted using the t.test() function (stats package) to compare colony biomass between *C. oleifera* plantations and fishpond habitats [30]. This approach measures the difference in group means relative to within-group variability and provides key statistics, including the t-value (indicating the magnitude of the difference), degrees of freedom (*df*) (reflecting sample size and group independence), and *p*-value (assessing statistical significance).

Spearman correlation coefficients were calculated to assess the monotonic relationships between ant colony quantity and nest structure parameters [31]. This non-parametric approach, implemented using the cor() function (stats package), is suitable for data that may not follow a normal distribution.

Finally, a random forest model was implemented using the randomForest package [32] to quantify the relative importance of meteorological variables in shaping colony structure. This approach provides a robust, tree-based assessment of variable importance and interactions.

## 3. Results

Our year-long monitoring of RIFA colonies revealed distinct structural and seasonal patterns across *C*. *oleifera* and fishpond habitats. Firstly, in the *C. oleifera* habitat, the annual density of RIFA was 25.36 nests per 667 m^2^, which was classified as a Level IV occurrence. Similarly, in the fishpond habitat, the annual occurrence density was 9.72 nests per 667 m^2^, which was also classified as Level IV. Worker ants dominated colony composition in both environments, accounting for 95.56% and 88.82% of populations, respectively, with peak abundances occurring during winter months. Notably, *C. oleifera* colonies maintained significantly larger biomass (6743 vs. 4440 ants/nest) and heavier workers (2.05 vs. 1.06 g/individual), while fishpond colonies exhibited higher larval densities (868 vs. 319 larvae/nest). Reproductive caste distributions differed markedly between habitats, with queens predominating in *C. oleifera* (30.35% of alates) versus winged females and larvae in fishponds (34.07%). Environmental analyses identified air pressure as the primary driver of worker abundance in both habitats, while precipitation most strongly influenced larval development stages.

### 3.1. Dynamic Pattern of RIFA Colony Structure in C. oleifera Habitat

**Figure 2:**
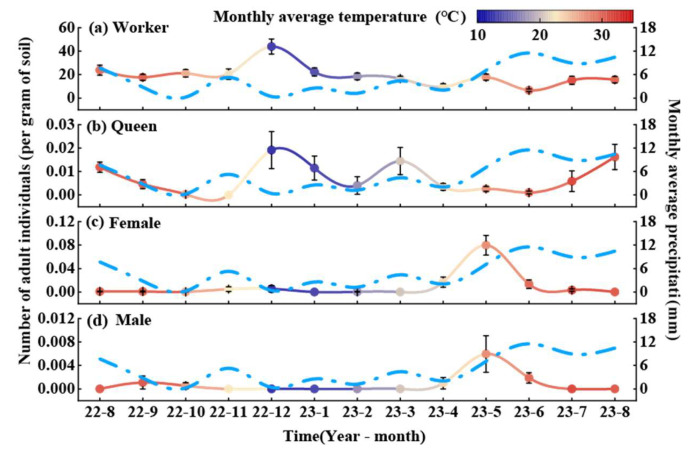
Dynamic pattern of adult RIFA in *C. oleifera* plantation. Values in the figure are mean ± standard error. The left *y*-axis indicates the number of RIFA adults (solid line), and the colors correspond to the average temperature of the month, with red indicating a high temperature in the month and dark blue indicating a low temperature; the right *y*-axis indicates the monthly precipitation (sky-blue dashed line); and the values in the graphs are the mean ± standard error. (**a**) Worker, (**b**) queen, (**c**) female, and (**d**) male.

Seasonal variation in the population structure of RIFA in the *C. oleifera* habitat showed regular fluctuations across castes (Figure 2). Worker abundance peaked in December 2022 and reached its lowest level in June 2023.

On average, the worker population stabilized at 19.15 ants per gram of soil throughout the year, with relatively minor fluctuations in other months (Figure 2a). The queen population reached its highest density in December at 0.019 ants per gram of soil. No queens were detected in November, and the annual average queen density was 0.0073 ants per gram of soil (Figure 2b). Females peaked in May, with no individuals detected in January, March, or August. The annual average density of females was 0.01 ants per gram of soil (Figure 2c). Similarly, males peaked in May at 0.006 ants per gram of soil, with no individuals observed in January, February, March, August, or November (Figure 2d).

**Figure 3:**
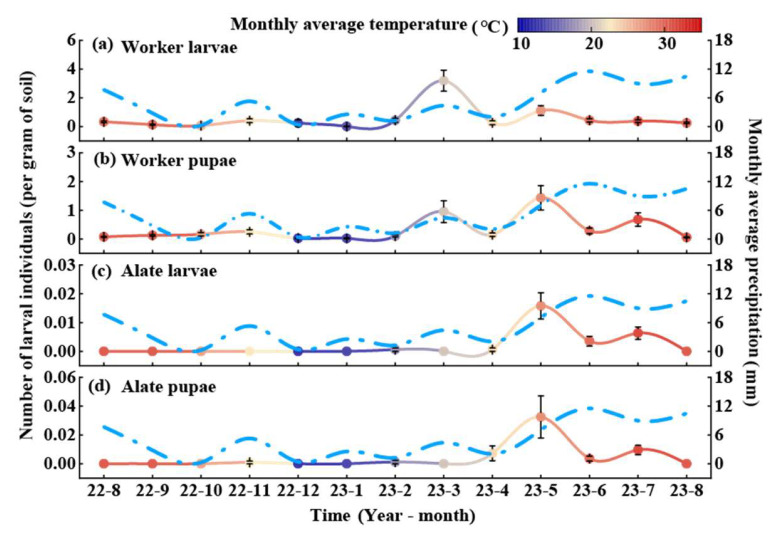
Dynamic pattern of larval and pupal RIFA caste stages in *C. oleifera* plantation. Values in the graphs are means ± standard errors. The left *y*-axis indicates the number of RIFA larvae (solid line), and the colors correspond to the average temperature of the month, with red indicating a high temperature in the month and dark blue indicating a low temperature; the right *y*-axis indicates the monthly precipitation (azure dashed line); and the values in the graphs are the mean ± standard error. (**a**) Worker larvae, (**b**) worker pupae, (**c**) alate larvae, and (**d**) alate pupae.

The larval population of RIFA also exhibited regular seasonal variations (Figure 3). Worker larvae peaked in March, with the lowest numbers recorded in January. The population remained relatively stable during the remaining months (Figure 3a). Worker pupae reached their highest density in May, while the lowest numbers were observed in January. The annual average density of worker pupae was 0.33 ants per gram of soil, with smooth variations in other months (Figure 3b). Alate larvae peaked in May, with no individuals detected from August to December or in January and March. The annual average density of reproductive larvae was 0.002 ants per gram of soil (Figure 3c). Alate pupae also peaked in May at 0.04 ants per gram of soil, with no individuals observed from August to December or in January and March (Figure 3d).

### 3.2. Dynamic Patterns of RIFA Colony Structure in Fishpond Habitat

**Figure 4:**
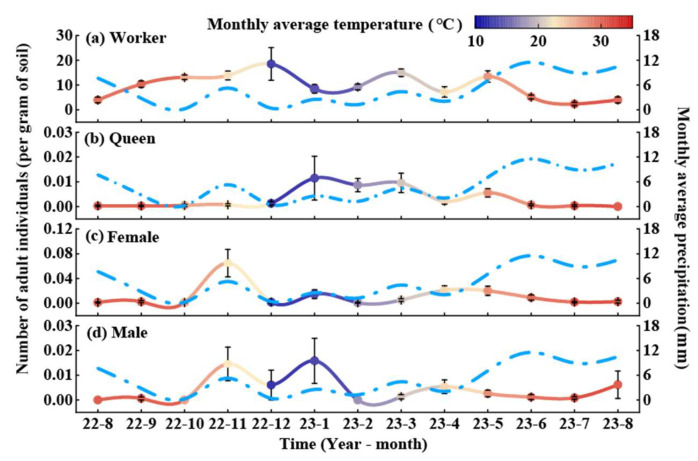
Dynamic patterns of RIFA adult infestation in fishpond habitat. Values in the figure are means ± standard errors. The left *y*-axis indicates the number of RIFA adults (solid line), and the colors correspond to the average temperature of the month, with red indicating high temperatures in the month and dark blue indicating low temperatures; the right *y*-axis indicates the amount of monthly precipitation (sky-blue dashed line); and the values in the graphs are means ± standard error. (**a**) Worker, (**b**) queen, (**c**) female, and (**d**) male.

The worker population peaked in December and dropped to its lowest level in July (Figure 4a). On average, the worker population stabilized at 9.59 ants per gram of soil throughout the year, with relatively gradual fluctuations in the remaining months. Queen density peaked in January. The absence of queens in August may be due to sampling constraints rather than actual absence from the colonies.

The annual average density of queens was 0.0032 ants per gram of soil (Figure 4b). Females showed a seasonal peak in November, while the lowest count was recorded in February (Figure 4c). Male reproductive ants peaked in January, with no individuals observed in February, August, or October. The annual average density of male ants was 0.004 ants per gram of soil (Figure 4d).

**Figure 5:**
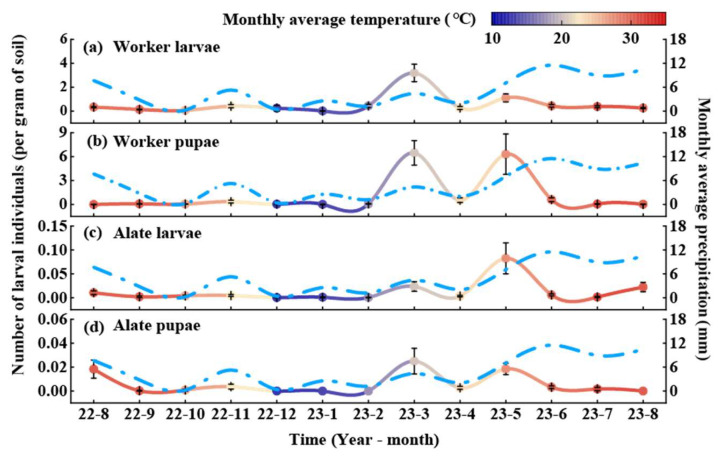
Dynamics of larval and pupal RIFA caste stages in fishpond habitat. Values are plotted as mean ± standard error. The left *y*-axis indicates the number of RIFA larvae (solid line), and the colors correspond to the average temperature of the month, with red indicating high temperatures during the month and dark blue indicating low temperatures; the right *y*-axis indicates the amount of monthly precipitation (sky-blue dashed line); and the values in the graphs are the mean ± standard error. (**a**) Worker larvae, (**b**) worker pupae, (**c**) alate larvae, and (**d**) alate pupae.

The populations of RIFA larvae and pupae also exhibited regular seasonal fluctuations (Figure 5). Worker larvae peaked in March, followed by a secondary peak in May. No larvae were detected in February. The annual average density of worker larvae was 0.08 larvae per gram of soil, with relatively stable numbers in other months (Figure 5a). Worker pupae reached their highest density in March, followed by a secondary peak in May, while the lowest count was observed in August. The annual average density of worker pupae was 1.13 pupae per gram of soil (Figure 5b). Alate larvae peaked in May (Figure 5c), while Alate pupae reached their highest density in March, followed by a secondary peak in May. No reproductive pupae were detected from January to February or from August to December. The annual average density of Alate pupae was 0.006 pupae per gram of soil (Figure 5d).

### 3.3. Dynamic Changes in the Proportion of Each RIFA Caste Stage

**Figure 6:**
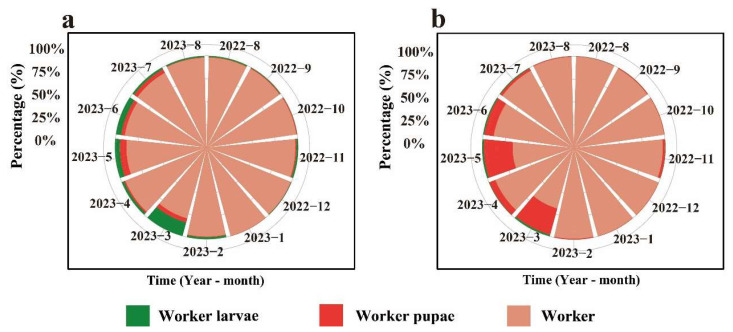
Dynamic pattern of the ratio of each RIFA caste stage of workers. (**a**) *C. oleifera* plantation habitat; (**b**) fishpond habitat. The figure uses colors to distinguish different developmental stages: green represents worker ant larvae, dark red represents worker ant pupae, and light red represents adult workers.

It can be observed that the proportion of workers in the *C. oleifera* habitat was the highest throughout the year. From March to July, during late spring and early summer, the proportion of worker larvae and pupae in the ant colony structure increased. Similarly, in the fishpond habitat, during the same period, the proportion of worker pupae was higher compared to other times (Figure 6).

In the *C. oleifera* habitat, the annual proportion of adult workers was 95.56%, that of worker larvae was 2.79%, and that of worker pupae was 1.66%. Among them, in March, the proportion of workers was 80.03%, while the proportion of worker larvae (15.38%) was higher than that of worker pupae (4.59%) (Figure 6a). In the fishpond habitat, the annual proportion of adult workers was 88.82%, that of worker larvae was 0.72%, and that of worker pupae was 10.46%. Among them, in March, the proportion of workers was 68.47%, while the proportion of worker pupae (28.59%) was higher than that of worker larvae (1.93%) (Figure 6b).

**Figure 7:**
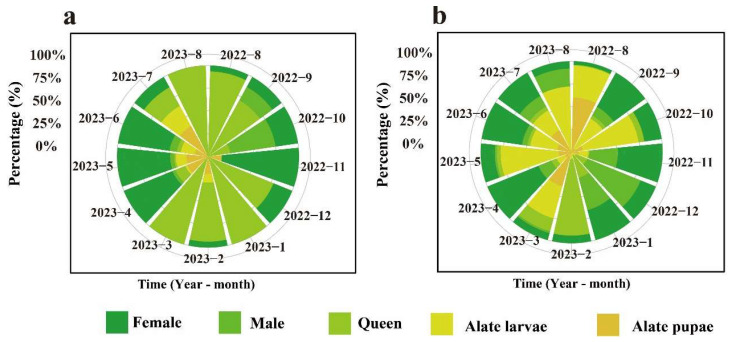
Seasonal dynamics in the proportions of alate stages in the RIFA colony. (**a**) *C. oleifera* plantation habitat; (**b**) fishpond habitat. The figure uses colors to distinguish between individuals of different castes: dark green represents females, light green represents males, yellow-green represents queens, light yellow represents alate larvae, and brown represents alate pupae.

According to the results of the annual variation in the ratio of alate ants in different developmental stages (Figure 7), it can be observed that in the *C. oleifera* habitat, the alate ants were primarily dominated by females and queens. In the fishpond habitat, the highest proportion of alate ants was represented by females and alate larvae.

In the *C. oleifera* plantation habitat, the annual proportion of queens was 30.35%, that of females was 40.23%, that of males was 3.33%, that of alate larvae was 8.56%, and that of alate pupae was 17.53%. The highest proportion of queens was observed in January, March, and August. The highest proportion of females was observed in November (0.85%), and the highest proportion of males was observed in September (16.79%). In July, the proportion of alate larvae was 25.23%, and the proportion of alate pupae was 38.37% (Figure 7a).

In the fishpond habitat, the annual proportion of queens was 8.57%, that of females was 30.56%, that of males was 11.30%, that of alate larvae was 34.07%, and that of alate pupae was 15.50%. The highest proportion of queens was observed in February. Furthermore, the highest proportion of females was observed in November (0.73%), and the highest proportion of males was observed in August (63.18%). In August, the proportion of alate larvae was 72.11%, and in March, the proportion of alate pupae was 38.76% (Figure 7b).

### 3.4. Differences in Colony Size, Nest Size, and Worker Characteristics of RIFA in Different Habitats

**Figure 8:**
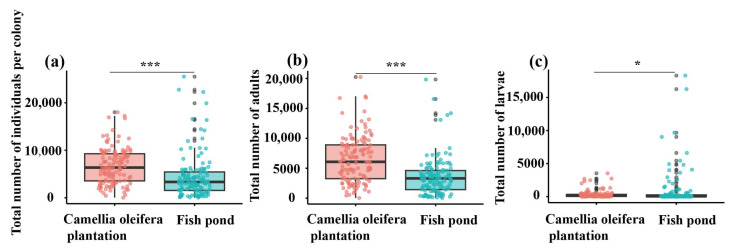
Differences in the number of ant colonies in different habitats. (**a**) Total number of ants in the colony, (**b**) total number of adult ants, and (**c**) total number of larvae, *n* = 130. The pink and red colors in the figure represent the *C. oleifera* plantation habitat, while the sky-blue color represents the fishpond habitat. The horizontal lines indicate the median, with whiskers extending to the maximum and minimum values. Data were analyzed using independent *t*-tests, and significance levels are denoted by asterisks (* *p* < 0.05 and *** *p* < 0.001).

This study aims to explore the differences in RIFA colony numbers in different habitats, specifically by comparing the total colony biomass, worker population, and larval quantity between *C. oleifera* and fishpond habitats (Figure 8). The total colony biomass in *C. oleifera* averaged 6743.09 ants per nest throughout the year, which was significantly higher than the fishpond habitat’s average of 4440.64 ants per nest (t = 4.39, df = 258; *p* < 0.001) (Figure 8a).

The total colony biomass in *C. oleifera* was significantly higher than that in the fishpond habitat, with mean values of 6743.09 ants per nest and 4440.64 ants per nest, respectively (t = 6.89, df = 258; *p* < 0.001) (Figure 8b). In contrast to the previous two indicators, the larval quantity in *C. oleifera* (mean of 318.92 ants per nest) was significantly lower than in the fishpond habitat (mean of 868.25 ants per nest) (t = −2.34, df = 258; *p* = 0.019) (Figure 8c).

**Figure 9:**
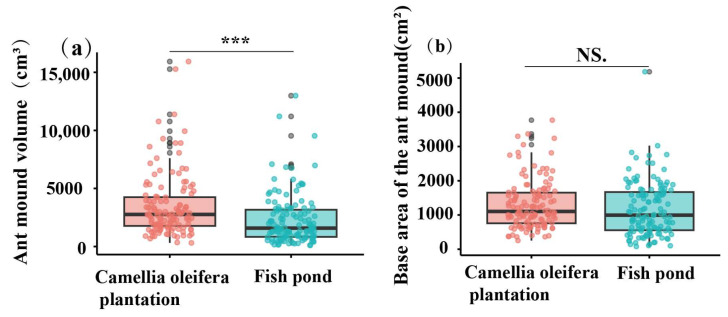
Differences in nest size across habitats: (**a**) volume of ant nests and (**b**) basal area of ant nests, *n* = 130. The figure depicts the *C. oleifera* plantation habitat in pink and red, while the fishpond habitat is represented by sky blue. The horizontal lines indicate the median, with whiskers extending to display the maximum and minimum values. The data were subjected to independent *t*-tests; asterisks (***) indicate the significance levels of *p*-values < 0.001 and “NS.” indicates no significant difference between habitats (*p* > 0.05).

The research results revealed that the average volume of ant nests in the *C. oleifera* habitat throughout the year was 318.92 cm^3^, which is significantly higher than the 2295.40 cm^3^ observed in the fishpond habitat (t = 3.86, df = 258; *p* < 0.001) (Figure 9a). The average basal area of ant nests in the *C. oleifera* habitat was 1301.73 cm^2^, while in the fishpond habitat, it was 1174.86 cm^2^. The difference in basal area between the two habitats was not significant (t = 1.36, df = 258; *p* = 0.17) (Figure 9b).

**Figure 10:**
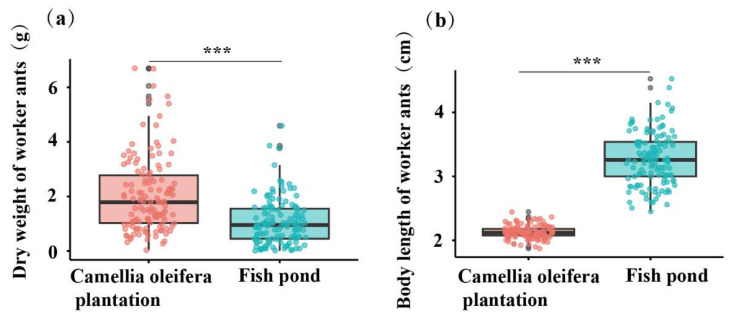
Differences in worker characteristics across different habitats: (**a**) average dry weight of workers and (**b**) average body length of workers, *n* = 130. The figure illustrates the *C. oleifera* plantation habitat depicted in shades of pink and red, while the fishpond habitat is represented by a sky-blue color. Horizontal lines represent the median, with whiskers extending to indicate the range from the maximum to the minimum values. Data were subjected to independent *t*-tests, and asterisks (***) denote the significance levels of the *p*-values < 0.001.

The research findings indicate that in the *C. oleifera* habitat, the average dry weight of workers throughout the year was 2.05 g per individual, which was significantly higher than the average of 1.06 g per individual observed in the fishpond habitat (t = 6.98, df = 258, *p* < 0.001) (Figure 10a). Furthermore, the average body length of workers in the *C. oleifera* habitat throughout the year was 2.13 cm per individual, which was significantly lower than the average of 3.28 cm per individual observed in fishpond habitats (t = −30.92, df = 258, *p* < 0.001) (Figure 10b).

### 3.5. Correlation Matrix Between Colony Size and Ant Nest Size

**Figure 11:**
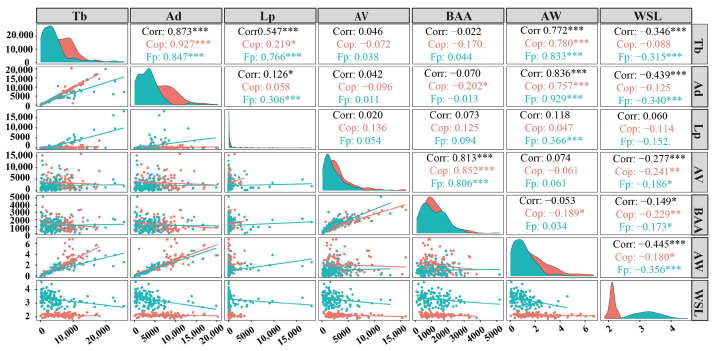
Relationship between ant colony quantity and ant nest size parameters. The colors represent Spearman correlation coefficients, where red indicates a positive correlation, blue indicates a negative correlation, and the intensity of the color reflects the strength of the correlation. The abbreviations used in the figure are defined as follows: total biomass of ant colonies (Tb), total number of workers (Ad), total number of larvae (Lp), volume of the ant nest (AV), basal area of the anthill (BAA), dry weight of the ant colonies (AW), body length of workers (WSL), correlation coefficient in the *C. oleifera* plantation (Cop), correlation coefficient in the fishpond (Fp), and overall correlation coefficient (Corr). The significance levels are denoted by asterisks, where * indicates *p* < 0.05, ** indicates *p* < 0.01, and *** indicates *p* < 0.001.

The relationship between the population of RIFA colonies and the parameters of ant nest size shows a significant correlation (Figure 11). Overall, there is a significant positive correlation (0.772, *p* < 0.001) between the total biomass of ant colonies (Tb) and the dry weight of the ants (AW). At the same time, there is a significant negative correlation (−0.346, *p* < 0.001) between the body length of workers (WSL) and the total biomass of the ant colonies. The total biomass of the ant colonies shows a positive correlation (r = 0.046) with the volume of the ant nest (AV) and a negative correlation (r = −0.022) with the basal area of the ant nest (BAA), but these correlations are not significant. There is a significant positive correlation (0.757, *p* < 0.001) between the total number of workers (Ad) and the dry weight of the ant colonies, and there is also a significant negative correlation (r = −0.439, *p* < 0.001) between the total number of workers and the volume of the ant nest, as well as the body length of the workers.

In the ecological environment of the *C. oleifera* plantation (Cop), there is a significant positive correlation (0.78, *p* < 0.001) between the total biomass of the ant colonies and the dry weight of the colonies. There is a negative correlation (r = −0.072, −0.170, −0.088) between the total biomass of the ant colonies and the volume of the ant nest, the basal area of the ant nest, and the body length of workers, respectively. However, there is a significant positive correlation (0.757, *p* < 0.001) between the total number of workers and the dry weight of the ant colonies. There is a negative correlation (−0.013) between the total number of workers and the basal area of the ant nest, and a negative correlation (−0.202) between the total number of workers and the body length of workers. The relationship with the basal area of the anthill reaches statistical significance (*p* < 0.05).

In the ecological environment of the fishpond (Fp), there is a significant positive correlation (0.833, *p* < 0.001) between the total biomass of the ant colonies and the dry weight of the colonies, and there is also a significant negative correlation (−0.315, *p* < 0.001) between the total biomass of the ant colonies and the body length of workers. The total biomass of the ant colonies shows a positive correlation (r = 0.038) with the volume of the ant nest and a positive correlation (r = 0.044) with the basal area of the ant nest. There is a significant positive correlation (0.93, *p* < 0.001) between the total number of workers and the dry weight of the ant colonies. Moreover, there are significant differences (*p* < 0.001) in the correlations between the total number of workers and the volume of the ant nest and the body length of workers (r = 0.011 and −0.34, respectively).

### 3.6. Correlation Analysis Between Meteorological Factors and Ant Nest Structure in Different Habitats

**Figure 12:**
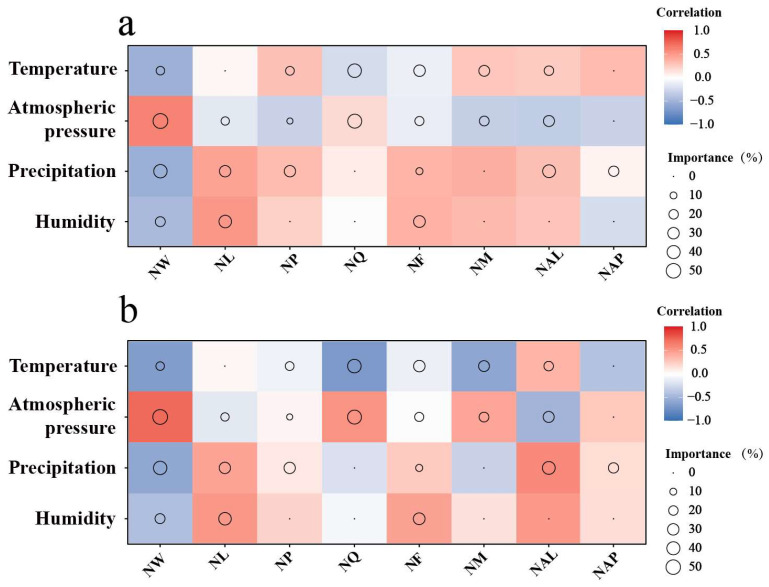
Variation in ant colony structure and its correlation with environmental factors in different habitats: (**a**) the *C. oleifera* plantation habitat and (**b**) the fishpond habitat. Different colors represent Spearman correlations, with red indicating a positive correlation and blue indicating a negative correlation. The darker the color, the stronger the correlation. The size of the circles represents the percentage of importance of each environmental variable, with larger circles indicating higher variable importance. The abbreviations used in the figure are explained as follows: number of workers (NW), number of worker larvae (NL), number of worker pupae (NP), number of queens (NQ), number of females (NF), number of males (NM), number of alate larvae (NAL), and number of alate pupae (NAP).

The Spearman correlation matrix and random forest (RF) modeling analysis results indicate significant effects of environmental factors on ant colony structure in different habitats (Figure 12).

In the *C. oleifera* habitat (Figure 12a), there is a significant positive correlation between air pressure and the number of workers, with a correlation coefficient of 0.6. Air pressure accounts for 42.93% of the variation in the number of workers. Temperature is the second most influential factor, explaining 35.32% of the variation, and it shows a negative correlation with worker numbers, suggesting that higher temperatures may inhibit the growth of worker populations. Precipitation (35.18%) and humidity (22.86%) have the greatest impact on worker larvae, and both show a positive correlation. Precipitation (40.92%) has the largest influence on the number of worker pupae. In contrast, the variation in the number of queens is explained by the four environmental factors (air pressure, temperature, humidity, and precipitation) to a lesser extent, with all of them explaining less than 15% of the variation. This indicates that these environmental factors have limited effects on the queen numbers. Furthermore, the main environmental factors influencing the variation in the number of females and males are ranked as air pressure, precipitation, and temperature. The number of alate larvae and pupae is influenced by air pressure, precipitation, and temperature, with a positive correlation observed between the number of alate larvae and precipitation.

In the fishpond habitat (Figure 12b), there is a significant positive correlation between air pressure and the number of workers, with a correlation coefficient of 0.73. Air pressure accounts for 51.80% of the variation in worker numbers. Precipitation and temperature are also important variables, especially precipitation, which explains 42.06% of the variation and shows a significant negative correlation (−0.64) with worker numbers. Humidity (36.50%) and precipitation (29.73%) have the greatest impact on worker larvae, and both show a positive correlation. Precipitation (29.40%) has the largest influence on the number of worker pupae. For the queen numbers, air pressure and temperature have the most significant effects, explaining 45.76% and 42.99% of the variation, respectively. Humidity and temperature explain 31.84% and 30.41% of the variation in the number of alate females, respectively. For males, humidity and temperature explain 26.84% and 21.43% of the variation in their numbers, respectively. Precipitation explains 38.28% and 23.85% of the variation in the number of winged ant larvae and pupae, respectively, and both show a positive correlation with precipitation.

## 4. Discussion

The colony structure of the RIFA shows marked seasonal fluctuations across caste and developmental stages, reflecting a finely tuned response to environmental variation. Based on a year-long study of two typical habitats—*C. oleifera* plantations and fishponds in South China—we found that worker ants peaked in abundance during winter (December to January), while reproductive ants were most abundant from spring to early summer (March to May). This pattern indicates a clear seasonal alternation in colony composition. Further analysis using Spearman correlation and random forest modeling revealed that worker abundance was primarily regulated by atmospheric pressure and temperature. In both habitats, pressure emerged as the strongest positive predictor, explaining 51.80% and 42.93% of the variation, respectively. In contrast, temperature was significantly negatively associated with worker numbers, suggesting that high temperatures may constrain colony expansion. Meanwhile, precipitation and humidity were key drivers during the brood-rearing phase, showing strong positive associations with the number of larvae and pupae of both worker and reproductive castes. These results not only validate the seasonal dynamics of RIFA colony structure based on field observations but also highlight caste-specific responses to climatic factors. Such adaptive strategies enable RIFA to synchronize development with environmental conditions, contributing to its invasive success and persistent spread across diverse habitats.

The winter increase in worker ants may result from favorable climatic conditions, such as stable temperatures, which enhance foraging efficiency and support energy acquisition for daily colony maintenance. In contrast, worker numbers drop significantly in summer, likely due to heat stress and limited resource availability. To cope with these environmental challenges, colonies may adopt behavioral adaptations such as relocating nests to shaded or sheltered areas, deepening nest structures, or enhancing ventilation to reduce thermal stress [33,34]. These behavioral engineering strategies are especially critical in fishpond habitats, where high temperatures and microclimatic instability prevail, enabling colonies to maintain baseline activity during the summer months. This pattern aligns with Porter’s (1988) findings that RIFA growth peaks at 31–32 °C, while higher temperatures inhibit worker activity and colony development [35]. Reproductive individuals—queens, females, and males—emerge primarily from spring to early summer, reflecting a distinct seasonal pattern of reproduction in subtropical regions. As temperature and humidity increase in tandem, colonies initiate nuptial flights and mating behaviors [36], accompanied by a sharp rise in the production of alate larvae and pupae, indicating a synchronized investment in reproductive development [15,37,38]. During this period, the number of worker larvae and pupae also peaks, marking an intensive phase of brood rearing and energy storage in preparation for dispersal events [39,40,41]. By late summer, larval numbers decline markedly, potentially due to reduced oviposition by queens or heat-induced developmental constraints on embryos [10,35,40].

Further quantitative analyses indicate that precipitation and humidity are key environmental drivers regulating the brood-rearing phase of RIFA. Both factors positively influence the number of larvae and pupae across worker and reproductive castes, a pattern consistent with the species’ broader distribution in humid regions [42]. Notably, caste-specific responses to environmental conditions exhibit strong habitat dependence, reflecting functional differentiation among castes. Previous studies have demonstrated that queen development is particularly sensitive to temperature and humidity variation, suggesting adaptive developmental preferences [43]. In our study, atmospheric pressure and temperature explained up to 45.76% and 42.99% of the variation in queen abundance, respectively, in fishpond habitats characterized by high humidity. In contrast, their explanatory power was below 15% in the drier Camellia plantation, suggesting substantial spatial heterogeneity in queen development across habitats. Furthermore, alate females and males demonstrated heightened sensitivity to changes in humidity and precipitation. Transcriptomic studies have revealed caste-specific expression of proteins involved in environmental sensing and developmental regulation [44]. Together, these findings support the view that RIFA employs finely tuned, caste-dependent regulatory mechanisms to adapt to multifactorial climatic conditions. Such ecological plasticity likely underpins its invasive success and capacity to maintain population expansion across diverse habitat types [45].

While this study shows that reproductive and brood care activities are concentrated in spring, there are regional differences. For example, a study in central Yunnan found that the peak period for Alate ants occurred from June to August, with the highest egg and larval numbers in August [46]. Similarly, a survey in southern China identified both spring and autumn as the most active seasons for RIFA, with the lowest activity during winter [15]. These discrepancies may arise from several factors. First, the study areas—*C. oleifera* plantations (25.36 nests/667 m^2^) and fishponds (9.72 nests/667 m^2^)—are high-density nest areas, which could significantly influence colony structure and developmental state. Second, unlike previous studies, this research was conducted nearly 20 years after RIFA’s invasion of southern China, suggesting that long-term adaptation and niche reshaping may have altered colony structure and behavioral strategies, which may not follow early invasion patterns. Moreover, previous studies suggest that geographic factors such as temperature, humidity, and local climatic conditions (e.g., latitude and elevation) significantly influence reproductive cycles, worker activity, and colony performance [46,47,48,49,50]. For instance, colonies in temperate regions exhibit more balanced activity, whereas those in regions with extreme temperature or humidity fluctuations may require adaptive strategies such as nest relocation or altered foraging behaviors. Human activities, including urbanization, have also driven differences in RIFA colony structures across habitats [50,51,52]. Over the past two decades, accelerated urban expansion and infrastructure development have significantly altered regional land-use patterns, particularly through the creation of more open and disturbed habitats such as farmlands, horticultural green spaces, and roadside edges [53]. These habitat changes offer RIFA new ecological niches. In agricultural ecosystems, particularly in structurally uniform, exposed environments like tea plantations and farmlands, RIFA has been able to establish stable colonies more easily [54,55]. Additionally, the widespread use of pesticides in agriculture may inadvertently provide a competitive advantage for RIFA, as it exhibits strong resistance to many common pesticides [56,57]. Collectively, climate change, land reclamation, and chemical interference are factors that have likely accelerated the spread and niche expansion of RIFA, and these should be considered in future invasion ecology studies and regional control strategies.

The absence of natural enemies is also a critical factor in the accelerated spread of RIFA. In its native range in South America, RIFA faces numerous natural enemies, including microsporidia, nematodes, viruses, phorid flies, parasitic wasps, mites, and other parasitic ants [58,59,60,61,62]. However, reports of natural enemies in China are scarce. In our study areas (*C. oleifera* plantations with 25.36 nests/667 m^2^ and fishponds with 9.72 nests/667 m^2^), the high nest density may suppress natural enemy populations, further exacerbating RIFA’s spread. The lack of natural enemies accelerates colony growth and spread, contributing to the difficulty of controlling its population. Therefore, both the absence of natural enemies and high-density populations likely interact to drive RIFA’s rapid expansion in China.

Finally, the social structure of RIFA may significantly influence its colony dynamics. Research has shown that RIFA exhibits two social forms—monogyne and polygyne—which differ significantly in terms of reproductive allocation, worker regulation, and dispersal capacity [11,63,64]. These forms also exhibit differences in gene flow, nest complexity, and task distribution within the colony [11,65]. Social structure may also be influenced by the biased inheritance of social chromosomes and the genetic composition of worker ants, which further complicates the relationship between social form and colony dynamics [66,67]. These differences affect task distribution within the colony and may enhance adaptability and expansion in various environments [47]. Polygyne colonies typically have higher nest densities and stronger colonization potential [1,68,69], which may partly explain the high nest density and relatively stable colony structure observed in the *C. oleifera* plantation area of this study. While this study did not directly assess social structure, its potential role in colony configuration and population regulation warrants further investigation.

The variation in resource availability and quality across different habitats also plays a crucial role in shaping RIFA’s colony structure and developmental strategies [3,70,71,72]. Camellia plantations, as a typical monoculture system, have sparse understory vegetation and relatively stable but homogeneous resource input. In contrast, fishponds often border water bodies and mixed vegetation, offering a more diverse food supply, including aquatic insects, detritus, and human food scraps. These resource differences likely influence the colony’s energy acquisition and allocation patterns, thereby regulating the proportional structure of various developmental stages. For instance, the fishpond’s heterogeneous environment may encourage colonies to invest more in reproductive individuals during peak nutrient availability, while the more stable but resource-poor *C. oleifera* plantations may prioritize maintaining a higher proportion of workers for daily energy input and nest maintenance. RIFA’s nutritional plasticity may be a key factor in its rapid adaptation and niche expansion across different habitats [73,74,75].

Climate change is likely to significantly impact RIFA distribution and colony dynamics, potentially expanding its invasive range into previously unsuitable habitats due to warmer temperatures and altered precipitation patterns. For instance, a recent analysis of new RIFA occurrences in China showed that regions with annual average temperatures around 18.6 °C and moderate annual precipitation (approximately 516.4 mm) are particularly vulnerable to invasion [44]. Similarly, studies in Virginia, USA, have documented a rapid northward expansion of RIFA, driven by warming temperatures and changing precipitation patterns, suggesting that climate change may significantly increase the ant’s invasive potential in previously unsuitable areas [76]. This trend is further supported by distribution modeling in China, which indicates that suitable habitats for RIFA will likely expand northward under future climate scenarios, potentially exposing an additional 130,000 km^2^ of urban areas to infestation [13,42].

Given these findings, future research should prioritize the development of climate-resilient management strategies that can effectively respond to these changing invasion dynamics. One promising direction is the establishment of Early Detection and Rapid Response (EDRR) systems. This approach could involve real-time monitoring networks that leverage remote sensing, unmanned aerial vehicles (UAVs), and machine learning algorithms to rapidly detect new RIFA incursions [77]. For instance, a recent study developed a remote-controlled RIFA trap that significantly improves emergency trapping efficiency, providing a practical tool for early intervention [78]. Additionally, AI-based deep learning models, such as DenseNet201 and ResNet50, have shown high accuracy in detecting environmental changes and invasive species from UAV images, suggesting that similar approaches could be adapted for RIFA monitoring [79]. Furthermore, the use of multispectral and thermal imaging from UAV platforms has proven effective in identifying early-stage infections and stress responses in vegetation, which could be adapted to detect RIFA nests [80]. Similarly, engaging local stakeholders in RIFA monitoring and control efforts can enhance the effectiveness of management strategies and promote early detection, ultimately reducing the ecological footprint of invasive species [81,82].

In parallel, predictive modeling should be expanded to incorporate climate change projections, land-use changes, and anthropogenic disturbance to forecast RIFA spread under different future scenarios [54,83]. For example, recent studies have shown that RIFA is likely to expand its range in the United States, particularly in the lower Midwest, under future climate scenarios [76]. In Taiwan, researchers found that paddy fields, main roads, and warehouses are critical dispersal pathways for RIFA, highlighting the importance of integrating land-use data into predictive models [84]. Additionally, studies have demonstrated that bioclimatic variables such as temperature, precipitation, and soil moisture play crucial roles in determining the habitat suitability of invasive species [85]. Hypervolume niche modeling has also been used to assess global invasion risks, revealing that climate change can significantly expand the ecological niches of invasive pests [86].

Adaptive control strategies will be critical as well. This may involve Integrated Pest Management (IPM) approaches that are adaptable to shifting climate conditions, including the use of biological control agents, pheromone traps, and genetic disruption technologies [59]. For example, new remote-controlled RIFA traps have been developed to improve emergency trapping efficiency, providing a practical tool for rapid response in high-risk areas [78]. Additionally, recent studies have demonstrated that certain herbal plants, such as *Tagetes lemmonii*, *Cymbopogon citratus*, and *Chrysopogon zizanioides*, can significantly reduce RIFA activity, offering an eco-friendly, sustainable alternative to chemical pesticides [87]. Pheromone-based monitoring and control systems also play a critical role in IPM strategies. For instance, the use of pheromone lures for mass trapping and mating disruption has been shown to effectively reduce pest populations in various agricultural systems, minimizing the need for chemical interventions [88]. Furthermore, ecological engineering and habitat manipulation can be employed to create unfavorable conditions for RIFA colonization, such as promoting native vegetation, enhancing soil health, and reducing habitat fragmentation, which have been proven effective in restoring ecological balance and reducing pest pressure [89]. Moreover, habitat restoration and connectivity should be prioritized to reduce the ecological footprint of RIFA and enhance the resilience of native ant communities. Long-term studies have shown that native ant populations can recover from RIFA invasion following habitat restoration, with significant improvements in species richness and community stability over decades [90]. In subtropical plantation forests, increased tree species richness has been found to suppress RIFA populations by enhancing native ant competition and improving soil microclimate, highlighting the importance of maintaining diverse plant communities for long-term pest ant control [91].

Finally, long-term ecological monitoring should be established to track the impacts of RIFA invasions on biodiversity, ecosystem function, and soil health. This could involve the creation of permanent monitoring plots, coupled with real-time climate and ecological monitoring, to continuously adjust management strategies in response to changing environmental conditions. By adopting these proactive strategies, we can improve the resilience of invaded ecosystems and reduce the long-term impacts of RIFA invasions.

## 5. Conclusions

This study demonstrated clear seasonal and habitat-based differences in the colony structure of the RIFA across *C. oleifera* plantations and fishpond habitats in South China. Worker abundance peaked in winter, while reproductive castes, including queens and alates, emerged primarily in spring and early summer. Larval and pupal stages also followed distinct seasonal trends in both habitats. Colony biomass, worker density, and worker body mass were significantly higher in *C. oleifera* plantations, while fishponds supported more larvae and alate males, suggesting habitat-driven reproductive allocation strategies. Colony structure proportions revealed a dominance of adult workers in both habitats, but a greater investment in brood (especially pupae) in the fishpond habitat. Significant correlations were found between colony size and nest characteristics. Worker body length was negatively associated with colony biomass and worker number, indicating a potential size–number trade-off. Environmental variables, particularly air pressure and temperature, strongly influenced colony composition, especially worker numbers and brood development. Overall, RIFA exhibits strong seasonal dynamics and ecological plasticity in response to habitat and environmental variation. These findings contribute to a better understanding of its invasive potential and provide a basis for developing habitat-specific monitoring and control strategies.

## Data Availability

The raw data and materials will be made available by the authors, without undue reservation, to any qualified researchers.
